# In severe liver disease, citrate can be used safely: the question remains—by which mechanism

**DOI:** 10.1186/s13054-020-2801-2

**Published:** 2020-02-24

**Authors:** Patrick M. Honore, Aurore Mugisha, Cristina David, Rachid Attou, Sebastien Redant, Andrea Gallerani, David De Bels

**Affiliations:** ICU Department, Centre Hospitalier Universitaire Brugmann-Brugmann University Hospital, Place Van Gehuchtenplein, 4, 1020 Brussels, Belgium

In a recent meta-analysis, Zhang and colleagues concluded that regional citrate anticoagulation (RCA) seems to be a safe anticoagulation method in liver failure patients undergoing continuous renal replacement therapy (CRRT) [1]. We would like to make some comments. Indeed, during RCA-CRRT, 30–70% of the administrated citrate can be removed by the dialyzer, and the remaining citrate enters the systemic circulation [1]. In the setting of severe liver dysfunction, citrate clearance is reduced by about 50%, which means liver failure patients are more susceptible to citrate accumulation [1]. Zhang et al. advocate the use of the “Khadzhynov rules” [2] to diagnose citrate accumulation in severe liver patients including the following diagnosis criteria: (i) decreased systemic ionized calcium (ionCa), (ii) increased demand for calcium substitution, (iii) elevated total calcium (totCa)/ionCa ratio, and (iv) metabolic acidosis. They conclude that most likely, the citrate accumulation incidences of the included studies were overestimated by only using the totCa/ionCa ratio [1, 2]. We somewhat disagree, as in the setting of citrate anticoagulation, with disturbed microvascular circulation causing altered hepatic function and citrate accumulation [3], it is our experience, like Klingele [3], that lactate better reflects citrate accumulation than calcium ratio and liver dysfunction itself [4]. Further, Zhang et al. state that theoretically, liver failure patients did not lose all of the liver citrate metabolization function [1]. Again, we do not totally agree. It is well known that in all cells, citrate can be metabolized within the Cori cycle (tricarboxylic acid cycle) [4], the linked metabolic pathways by which muscles, even in the absence of oxygen, remain capable of functioning, producing lactate. The Cori cycle is activated both by citrate [4] and adrenaline [4], the latter leading to the production of lactate [4]. The liver citrate anticoagulation threshold (L-CAT) trial showed the safety of CRRT-citrate in patients with severely impaired liver function [5]. We conclude that the Cori cycle is functional without oxygen [4] and that citrate metabolism is less dependent on the hepatic function itself and more dependent upon the whole microcirculation of the body, as suggested by Klingele, which makes lactate the best marker of liver failure associated with microcirculation failure. Whenever the liver is unable to metabolize citrate, the Cori cycle takes over the task of the failing liver [4].

## Authors’ response

We thank Patrick M Honore et al. for their valuable comments regarding our recent article [1].

First, Honore et al. disagreed with the “Khadzhynov rules” and advocated lactate could be a better predictor for citrate accumulation (CA). The most precise way to identify CA is the test of plasma citrate concentration, which is not routinely available during our clinical practice. Hetzel et al. [6] found out that the totCa/ionCa ratio was highly correlated with the citrate plasma level (*R* = 0.85; *P* < 0.001). However, the totCa/ionCa ratio may still not predict CA in all cases [2]. Schneider et al. [7] proposed a clear distinction between CA and net citrate overload, which were characterized by metabolic acidosis and metabolic alkalosis, respectively. Hypocalcemia is a sensitive indicator of CA, however, its specificity is inadequate [2]. Therefore, the clinical diagnosis of CA should base on comprehensive indicators including serum calcium, calcium ratio, acid-base status, and anion gap, which are the theoretical consequences of CA. Recent studies found out that serum lactate was an independent predictor of CA. As we know, hyperlactatemia could occur without the occurrence of hypoxia (type B). On the other hand, hyperlactatemia could result in metabolic acidosis, which was one of the CA diagnosis criteria reported by Khadzhynov et al. [2]. Therefore, we do not think the use of only lactate is a better diagnosis strategy of CA.

Second, the authors did not totally agree with us regarding the liver citrate metabolization function of patients with liver failure. Our statement was based on the theory that the ability of liver failure patients to metabolize citrate is not totally lost but decreased, resulted in an increased risk of CA [7]. Furthermore, we stated that extrahepatic organs with high amounts of mitochondria such as the skeletal muscle and kidney cortex could metabolize citrate as well. We agree with the authors that a potential inducible citrate metabolic pathway may exist outside the liver. However, to the best of our knowledge, there is insufficient evidence that the Cori cycle is functional without oxygen. As we know, the Cori cycle, where the citrate is metabolized ultimately, is an oxygen-dependent process [8]. We believe that the whole body microcirculation is most likely more important for citrate metabolism than liver function.

Sincerely,

Wei Zhang, Shiren Sun, Xiangmei Chen, and Ming Bai

## Data Availability

Not applicable.

## Abbreviations

CRRT: Continuous renal replacement therapy
ionCa: Ionized calcium
L-CAT: Liver citrate anticoagulation threshold
RCA: Regional citrate anticoagulation
totCa: Total calcium

## References

[1] Zhang W, Bai M, Yu Y, Li L, Zhao L, Sun S, Chen X (2019). Safety and efficacy of regional citrate anticoagulation for continuous renal replacement therapy in liver failure patients: a systematic review and meta-analysis. Crit Care.

[2] Khadzhynov D, Schelter C, Lieker I, Mika A, Staeck O, Neumayer HH (2014). Incidence and outcome of metabolic disarrangements consistent with citrate accumulation in critically ill patients undergoing continuous venovenous hemodialysis with regional citrate anticoagulation. J Crit Care.

[3] Klingele M, Stadler T, Fliser D, Speer T, Groesdonk HV, Raddatz A (2017). Long-term continuous renal replacement therapy and anticoagulation with citrate in critically ill patients with severe liver dysfunction. Crit Care.

[4] Honore PM, De Bels D, Redant S, Attou R, Kugener L, Boer W (2019). Inducible metabolic pathway for citrate metabolism in case of major liver dysfunction: fact or fiction?. Crit Care.

[5] Slowinski T, Morgera S, Joannidis M, Henneberg T, Stocker R, Helset E (2015). Safety and efficacy of regional citrate anticoagulation in continuous venovenous hemodialysis in the presence of liver failure: the liver citrate anticoagulation threshold (L-CAT) observational study. Crit Care.

[6] Hetzel GR, Taskaya G, Sucker C, Hennersdorf M, Grabensee B, Schmitz M (2006). Citrate plasma levels in patients under regional anticoagulation in continuous venovenous hemofiltration. Am J Kidney Dis.

[7] Schneider AG, Journois D, Rimmele T (2017). Complications of regional citrate anticoagulation: accumulation or overload?. Crit Care.

[8] Akram M (2014). Citric acid cycle and role of its intermediates in metabolism. Cell Biochem Biophys.

